# Regulation of ZFP36 by lncOlfr29 promotes inflammation through NLRP3

**DOI:** 10.3389/fimmu.2025.1642783

**Published:** 2025-08-26

**Authors:** Wenyue Cheng, Fan Li, Yuan Zhang, Yunhuan Gao, Rongcun Yang

**Affiliations:** ^1^ Department of Immunology, Nankai University School of Medicine, Nankai University, Tianjin, China; ^2^ Translational Medicine Institute, Tianjin Union Medical Center of Nankai University, Tianjin, China; ^3^ State Key Laboratory of Medicinal Chemical Biology, Nankai University, Tianjin, China

**Keywords:** macrophages, lncOlfr29, NLRP3, ZFP36, IL-1β

## Abstract

**Objective:**

The functional state of macrophages is regulated by multiple factors and closely related to the occurrence and development of various diseases. The aim of this study is to discover a new regulatory factor in macrophages, which can serve as a target for disease prevention and treatment.

**Methods:**

Long non-coding RNA (lncRNA) lncOlfr29 was discovered through RNA sequencing. The functions of lncOlfr29 were investigated by bioinformatics analysis, lncOlfr29 shRNA silencing and overexpressing adenovirus, and lncOlfr29 knockout (KO) mice. To investigate the function of lncOlfr29 *in vivo*, we also established a Salmonella infection model and DSS-mediated colitis using lncOlfr29 KO mice.

**Results:**

We here identified a novel lncRNA named lncOlfr29 in macrophages and demonstrated that lncOlfr29 promoted inflammation by enhancing NLRP3-mediated IL-1 β maturation and pyroptosis of macrophages. *In vivo* experiments showed that lncOlfr29 could promote resistance to Salmonella infection and sensitivity to DSS mediated colitis. Mechanistically, lncOlfr29 could bind to zinc finger protein 36 (ZFP36) to eliminate the degradation of ZFP36 on NLRP3 mRNA. Knockout of lncOlfr29 led to a decrease of NLRP3 in cytoplasm, reducing macrophage pyroptosis and IL-1 β maturation.

**Conclusion:**

Our data demonstrate that lncOlfr29 can regulate expression of NLRP3 through binding with ZFP36. These results will provide new insights into the treatment of inflammatory diseases.

## 1 Introduction

Macrophages, as an important component of the innate immune system, are widely present in the body. These cells exhibit complex and diverse functions in the body, such as regulating inflammation and the production of cytokines (1). It is worth noting that the functional status of macrophages is closely related to the occurrence of various diseases, including inflammatory bowel disease (IBD), cancer, autoimmune diseases, cardiovascular diseases, neurodegenerative diseases, metabolic diseases and trauma (1–3). However, how to control the functions of macrophages is still not completely clear.

The function of macrophages is controlled by multiple factors such as inflammasome. NLRP3 is one of the most representative inflammasomes. It consists of NOD-like receptor protein 3 (NLRP3), apoptosis-associated speck-like protein containing CARD (ASC) and pro-caspase-1 (4). NLRP3 can be activated by a series of stimuli, including microbial components, toxins, RNA viruses, ATP, mitochondrial reactive oxygen species, cholesterol and sodium urate (5, 6). When NLRP3 is activated, an assembly involving ASC and pre-caspase-1 occurs, resulting in the formation of the NLRP3 inflammasome complex. Then caspase-1 self-lyses to activate caspase-1 (7). The activated caspase-1 can lyse and mature the cytokine IL-1β and the pore-forming protein gasdermin D (GSDMD) (8, 9). Mature GSDMD causes the pyroptosis of macrophages, releases mature IL-1β and IL-18, and triggers an inflammatory response to infection (10, 11).

LncRNA is a larger transcript (about 200 nt), and its transcription mode is similar to that of mRNA, but it is not processed into proteins (12). Interestingly, lncRNAs can be engaged in direct physical interactions with other nucleic acids, proteins, lipids, or structural components (13). They plays a role in epigenetics, regulation of transcription factor activity, and cytogenetics (13). The overexpression, deficiency or mutation of lncRNAs has been implicated in numerous human diseases (14). Thus, lncRNAs are the potential targets for various diseases (15). Indeed, although lncRNA-targeted therapy has not yet achieved clinical transformation, due to the discovery of lncRNA and its role in diseases, research on using lncRNA as a drug target is ongoing (15). Since macrophages play a critical role in the inflammatory diseases (14), identification of a new lncRNA in the macrophages could produce important effects on therapy of these diseases. Here we found that lncOlfr29 in macrophages can promotes inflammation through enhancing expression of NLRP3. LncOlfr29 can control the expression of NLRP3 by binding to ZFP36, which can bind to the 3’UTR of NLRP3 mRNA to cause mRNA degradation (16).

## 2 Materials and methods

All key reagents, resources and oligoes were listed in **Supplementary Table 1** in **Supplementary Materials**.

### 2.1 Mice and cell lines

C57BL/6 mice were from by the Model Animal Research Center of Nanjing University. LncOlfr29 knockout (KO) mice on a C57BL/6J background were generated using CRISPR-Cas9 system by the Model Animal Research Center of Nanjing University (Nanjing, Jiangsu, China). NLRP3 knockout (-/-) mice were from Prof. Meng, Pasteur Institute, Shanghai. B6.SJL-CD45a(Ly5a) (CD45.1) mice were from the Model Animal Research Center of Beijing. All mice were maintained in specific pathogen-free (SPF) conditions in the Animal Center of Nankai University. Murine experiments were performed according to Nankai University Guide for the Care and Use of Laboratory Animals. Human macrophage cell line THP-1 was from the American Type Culture Collection.

### 2.2 Mouse models

Salmonella Typhimurium (S. T) infection was performed according to the previously reported method (17). Briefly, mice were first treated by gavage with 7.5 mg of streptomycin, and then infected with S. T (1 × 10^3^CFUs/mouse for chronic infection, 1 × 10^7^ CFUs/mouse for acute infection) at 20 hours after streptomycin treatment. Weight changes in mice was calculated as: % weight change = (weight at day X-day 0/weight at day 0) × 100. The colon and lung tissues were embedded in OCT compound for immuno-staining, or in paraffin for hematoxylin/eosin (H&E) staining. Spleen, Lung and liver were homogenized for 2 mins in PBS. CFUs were quantified after plating lysates onto LB agar for 24 hrs.

DSS-mediated colitis was carried out according to previously reported method (18). Briefly, mice (15 mice per group) were received 2.5% (w/v) DSS in their drinking water for 7 days, and then switched to regular drinking water. Body weights were detected and calculated as: % weight change = (weight at day X-day 0/weight at day 0) × 100. Mice were also investigated for rectal bleeding, diarrhea, and signs of morbidity such as hunched posture and failure to groom. Disease activity index (DAI) and histological evaluation were assessed according to methods (19, 20). DAI was the average of combined scores of stool consistency, bleeding and weight loss: Weight loss: 0, none; 1, 1–5%; 2, 5–10%; 3, 10–15%; 4, >15%; Bloods: 0, normal; 2, slight bleeding; 4, gross bleeding; Diarrhea: 0, normal; 2, loose stools; 4, watery diarrhea.

Histology scores (E + I) were assessed as followings: Infiltration (I), 0 = no infiltrate; 1 = infiltrate around the crypt basis; 2 = infiltrate reaching the lamina muscularis mucosae; 3 = extensive infiltration reaching the lamina muscularis mucosae and thickening of the mucosa with abundant edema; 4 = infiltration of the lamina submucosa; Epithelium (E): 0 = normal morphology; 1 = goblet cell loss; 2 = goblet cell loss in large areas; 3 =  crypt loss; 4 =  crypt loss in large areas.

For macrophage transplanted experiments. Bone marrow cells (BMCs) were collected from WT or *LncOlfr29-/-* mice, and then the monocytes/macrophages were isolated from BMCs through sorting after staining using CD11b antibody. Isolated monocytes/macrophages were then injected into different recipient mice, which were irradiated (8 Gy, a single dose) using a Shepherd Mark I Cesium Irradiator (J.L. Shepherd and Associates). After 3 weeks, DSS model was performed in the recipient mice.

### 2.3 Preparation of macrophages

Murine macrophages were generated from the peritoneal cavity of mice, which were intraperitoneally injected with 4 mL of 3% thioglycollate medium. The cells from the peritoneal cavity were seeded in RPMI containing 10% FBS. After removing non-adherent cells, adherent cells were collected.

For bone marrow derived macrophages (BMDMs), bone marrow cells were first obtained from bone marrow of the tibia and femur, and cultured for 6 days in RPMI with 10% FBS, 20 ng/ml mouse M-CSF and 1% penicillin/streptomycin.

For human peripheral blood cells derived macrophages (PBDMs), after collecting human peripheral blood, the cells were isolated using Percoll. Then isolated cells were cultured in RPMI containing 10% FBS. After removing non-adherent cells, adherent cells were cultured in the medium with GM-CSF and IL-4 for 5 days.

### 2.4 Construction and transduction of ShRNA or lncOlfr29 lentiviruses

Using BLOCK-iT™ RNAi Designer (Invitrogen), short hairpin RNA (shRNA) target sequences were selected. Through pGreenPuro™ cloning and expression lentivector kit, shRNA or lncOlfr29 constructs were generated according to the manual. The control luciferase control RNA is from the kit. For packaging lentivirus particles, shRNA, lncOlfr29 or control lentivector together with packaging plasmids pMD2.G and psPAX2 were co-transfected into 293T cells. Macrophages were infected by centrifugation with the lentivirus in the presence of 8 μg/ml polybrene.

### 2.5 *Ex vivo* stimulation

For inflammasome activation, macrophages were primed with LPS (2 μg/mL) for 4 h, and then stimulated with Dotap to activate caspase 11(mice)/caspase 4(human) or nigericin (5 μM) to activate NLRP3 inflammasome, DOTAP-transfected flagellin (5μg/mL) to activate NLRC4 inflammasome.

### 2.6 Actinomycin D chase assay

Macrophages transfected with ZFP36 siRNA (siZFP36) and exogenous lncOlfr29 (oeOlfr29) were treated with actinomycin D (0.5 mg/mL) for indicated time periods to initiate the time-course experiment. NLRP3 mRNA levels were detected by qRT-PCR.

### 2.7 RNA-FISH

RNA fluorescence *in situ* hybridization (RNA-FISH) was carried out according to reported protocol (21). Briefly, cells were first slicked on 0.01% poly-lysine-treated slides. For cell membrane perforation, cells were processed with CSK+0.4% Triton X-100 buffer for 30 s. Pre-warmed 5% goat serum was used for blocking for 30 mins at 37°C. The slides incubated with primary antibody at 37°C for 1 hour. The slides were incubated with secondary antibody at 37°C for 30 mins after washing. and then dehydrated through ethanol series (85, 95, and 100% ethanol), and hybridized using the indicated probes overnight at 37°C in a humid chamber. After washing, DAPI dye was added.

### 2.8 RNA immunoprecipitation

RNA immunoprecipitation (RIP) was carried out according to reported protocol (22). Briefly, cells were first added into ice cold IP lysis buffer containing 0.5% ribonuclease inhibitor, and incubated on ice for 5 mins with mixing. The lysates were centrifuged at 13,000 g for 10 mins to pellet the cell debris at 4°C. For preclearing, protein G agarose was added and incubated for 1 hour at 4°C with rotation. After that, immune-precipitating antibodies were added and incubated overnight at 4°C with rotation. Then protein G agarose was pelleted and washed with IP lysis buffer, containing 0.5% ribonuclease inhibitor. Finally, RNA was extracted from the complexes of protein/RNA and quantified by qPCR.

### 2.9 Fractionation of cytoplasm and nucleus

The cells were first incubated with hypotonic buffer, containing 25 mM Tris-HCl, pH 7.4, 1 mM MgCl2, 5 mM KCl on ice for 5 min, and then an equal volume of hypotonic buffer containing 1% NP-40 was added. The supernatants were collected as the cytosolic fraction after centrifugation at 5000 × g for 5 mins. The pellet was re-suspended in buffer, containing 20 mM HEPES, pH 7.9, 400 mM NaCl, 1 mM EDTA, 1 mM EGTA, 1 mM DTT and1 mM PMSF, and then incubated at 4°C for 30 mins. Nuclear fraction was collected by centrifugation at 12,000 g for 10 mins.

### 2.10 Others

Total RNA extraction, qRT-PCR, Western blotting, cell isolation, flow cytometry and immunoprecipitation (IP) was performed according to our previously method (22, 23).

### 2.11 Statistical analyses

ONE-way ANOVA Bonferroni’s Multiple Comparison text and two side student’s t-test were used. The survival curves were compared using the generalized Wilcoxon’s test. All of these were performed by GraphPad Prism 5 software. A 95% confidence interval was considered as significance and defined as p <0.05.

## 3 Results

### 3.1 LncOlfr29 expression in macrophages

**Figure 1:**
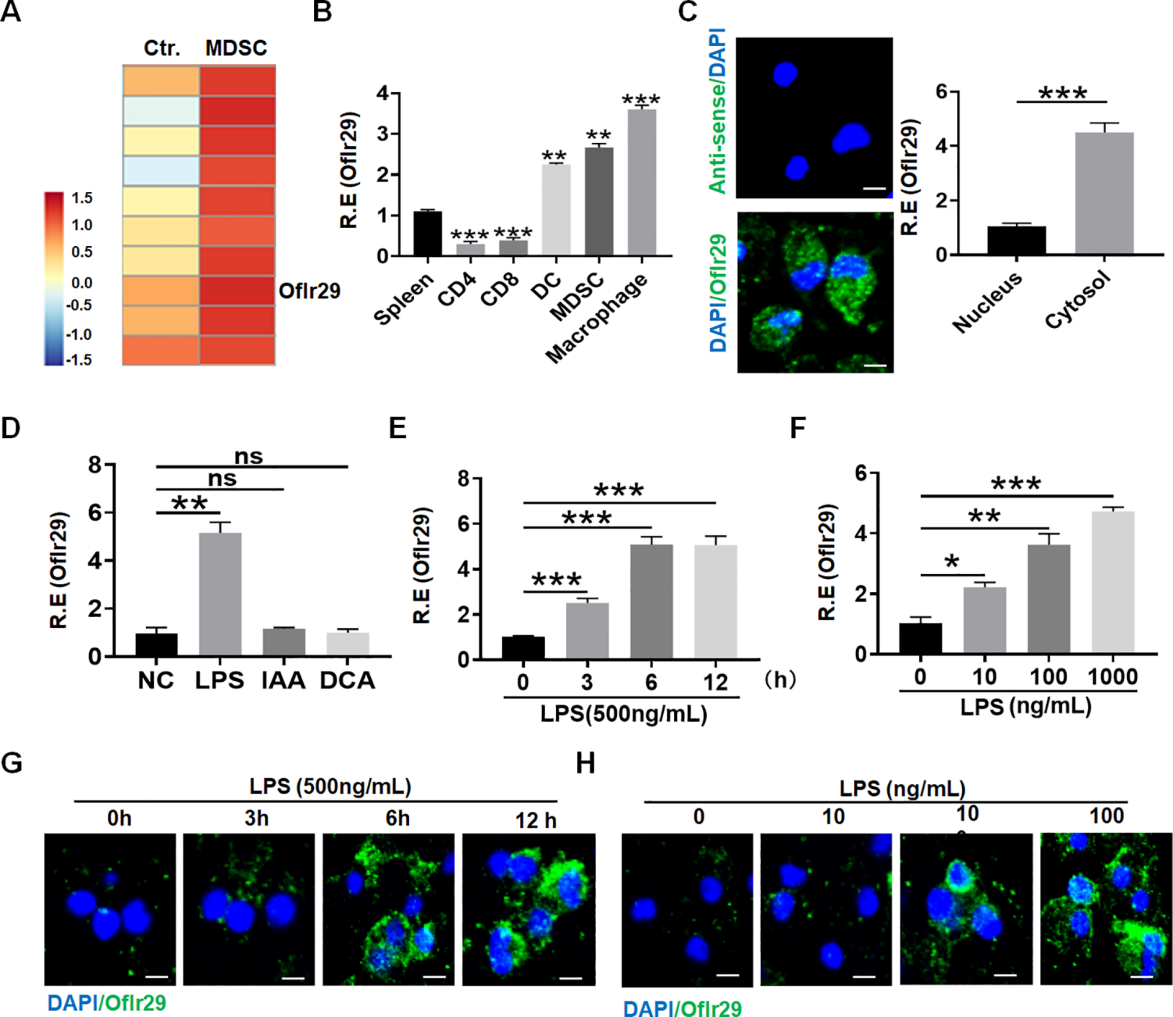
LncOlfr29 expression in macrophages. **(A)** Microarray of lncOlfr29 in myeloid-derived suppressor cells (MDSCs). MDSCs were generated, and lncRNA expression was evaluated using a lncRNA expression microarray. **(B)** QRT-PCR of lncOlfr29 in spleen, CD4, CD8, dendritic cells (DC), MDSCs and macrophages sorted from spleen by flow cytometry. R. E, relative expression. **(C)** RNA-FISH of lncOlfr29 in mouse macrophages and qRT-PCR of lncOlfr29 in the cytosol and nucleus. Nuclei were stained with DAPI (blue); Green, lncOlfr29. Scale bar, 2.5 μM. **(D)** LncOlfr29 expression in macrophages upon exposure to gut microbiota derived factors LPS (100 μg/ml), IAA (1μg/ml) and DCA (1μg/ml). NC, medium only. **(E, F)** QPCR of lncOlfr29 in the macrophages upon exposure to LPS at different times and concentrations. **(G, H)** RNA-FISH of lncOlfr29 in the macrophages upon exposure to LPS at different times and concentrations. Scale bar, 2.5 μM. Data were shown by mean ± SD; Student’s *t*-test; Ns, no significance; *p<0.05;**p<0.01;***p<0.001.

To discover a new factor that can regulate the function of macrophages, we reanalyzed the previous RNA sequencing data (17, 24). Data showed that lncRNA Olfr29-ps1 (lncOlfr29) was significantly highly expressed in myeloid derived suppressor cells (MDSCs) (**Figure 1A**). Compared with other immune cells, the expression of lncOlfr29 in mouse macrophages was significantly higher (**Figure 1B**). This lncOlfr29 could be detected in both the cytoplasm and the nucleus, but it was more common in the cytoplasm of macrophages (**Figure 1C**). Bioinformatics analyses revealed that lncOlfr29 was located in the chromosome 4 in mice and chromosome 16 in human (**Supplementary Figure 1**). There had widely epigenetic modification on the promoter region of lncOlfr29 in the macrophages not only in human but also in mice, which could promote the expression of genes (**Supplementary Figure 1**), supporting the high expression of this lncRNA in macrophages. We also sought out the factor(s) that might affect the expression of lncOlfr29. Since gut microbiota plays a critical role in human healthy and diseases (25), we observed the effects of several metabolites from the gut microbiota on the expression of lncOlfr29 in the macrophages. Data showed that LPS could significantly promote the expression of lncOlfr29 (**Figure 1D**). Furthermore, LPS mediated lncOlfr29 was time- and dose-dependent (**Figures 1E–H**). The expression level of lncOlfr29 was the highest 5 hours after LPS stimulation, and the expression level of lncOlfr29 increased with the increase of LPS concentration (**Figures 1E–H**). Thus, we identify a new lncRNA lncOlfr29 in macrophages, which can be promoted by LPS.

### 3.2 LncOlfr29 promotes pyroptosis of macrophages and maturation of IL-1β

**Figure 2:**
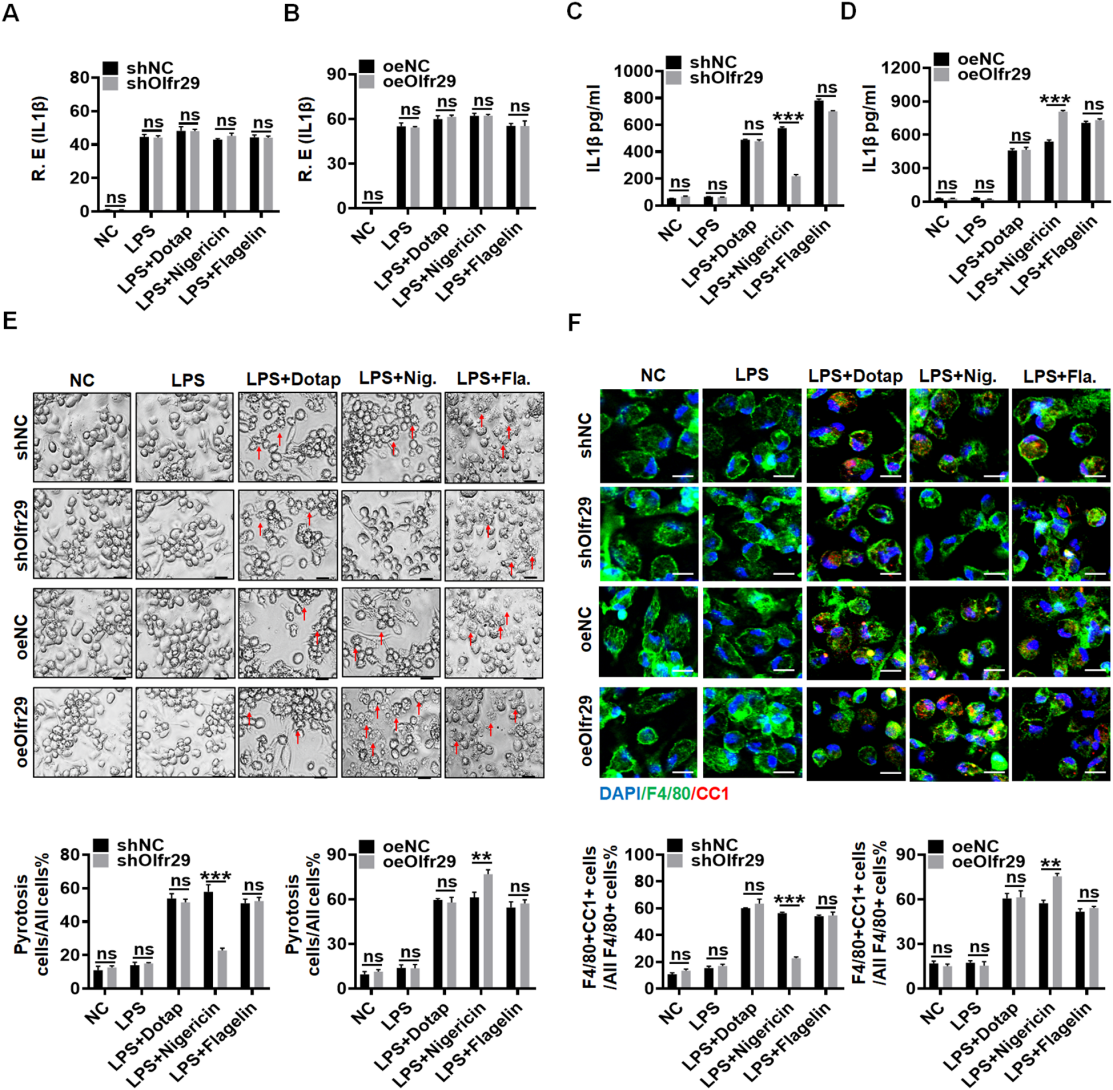
LncOlfr29 promotes pyroptosis of macrophages and maturation of IL-1β. **(A, B)** QRT-PCR of IL-1β in lncOlfr29 shRNA (shOlfr29) and exogenous lncOlfr29 (oeOlfr29) transfected macrophages. **(C**, **D)** ELISA of mature IL-1β in the supernatants of shOlfr29 and oeOlfr29 transfected macrophages. **(E)** Observation under light microscope for shOlfr29 and oeOlfr29 transfected macrophages. Arrows, typical pyroptosis cells. **(F)** Immuno-staining of cleaved caspase-1 (CC-1) in shOlfr29 and oeOlfr29 transfected macrophages. The macrophages were isolated from the peritoneal cavity of thioglycollate-treated mice, and stimulated by LPS, LPS + Dotap, LPS + nigericin or LPS + flagellin. NC, medium only. ShNC, shRNA control; OeNC, oeOlfr29 control. Data were shown by mean ± SD; Student’s *t*-test; Ns, no significance; **p<0.01;***p<0.001.

Next, we studied the role of lncOlfr29 in macrophages. To this end, we first generated lncOlfr29 silencing (lncOlfr29 shRNA) and lncOlfr29 overexpressing (exogenous lncOlfr29) lentiviruses, both of which demonstrated significant effects on lncOlfr29 expression levels (**Supplementary Figure 2**) Then macrophages were transfected with these lentivirus, and stimulated with multiple ligands, which could activate the inflammasomes, including LPS, LPS plus Dotap (activator of caspase-11) (26), LPS with flagellin (activator of NLRC4) (27), LPS with nigericin (activator of NLRP3) (28, 29). Data showed that NLRP3 ligand LPS plus nigericin significantly reduced the production of mature IL-1β (mIL-1β) but not IL-1β mRNA in lncOlfr29 silenced macrophages, while LPS plus Dotap or LPS plus flagellin had no significant effects (**Figures 2A, C**). In lncOlfr29 overexpressed macrophages, mIL-1β was significantly increased in response to LPS plus nigericin, but not to LPS plus Dotap or LPS plus flagellin (**Figures 2B, D**). Since IL-1β maturation often company with pyroptosis (8, 9), which is a lytic cell death induced by pathogen infection or endogenous challenge (30), we also observed the effects of silencing or overexpressing lncOlfr29 on the pyroptosis. Silencing lncOlfr29 could significantly alleviate LPS plus nigericin mediated pyroptosis on macrophages, but LPS plus Dotap or LPS plus flagellin mediated pyroptosis was not affected (**Figure 2E**). The pyroptosis of lncOlfr29 overexpressed macrophages was also more severe upon exposure to LPS plus nigericin than to LPS plus Dotap or LPS plus flagellin (**Figure 2E**). Immunofluorescence analysis of cleaved caspase-1 (CC-1) for detecting pyroptosis (31, 32) also showed that silencing lncOlfr29 could alleviate pyroptosis, while overexpression of lncOlfr29 aggravated pyroptosis (**Figure 2F**). Thus, lncOlfr29 can promote the pyroptosis of macrophages and the maturation of IL-1β upon exposure to NLRP3 ligand.

### 3.3 LncOlfr29 and NLRP3 KO mice have similar functions in the macrophages

**Figure 3:**
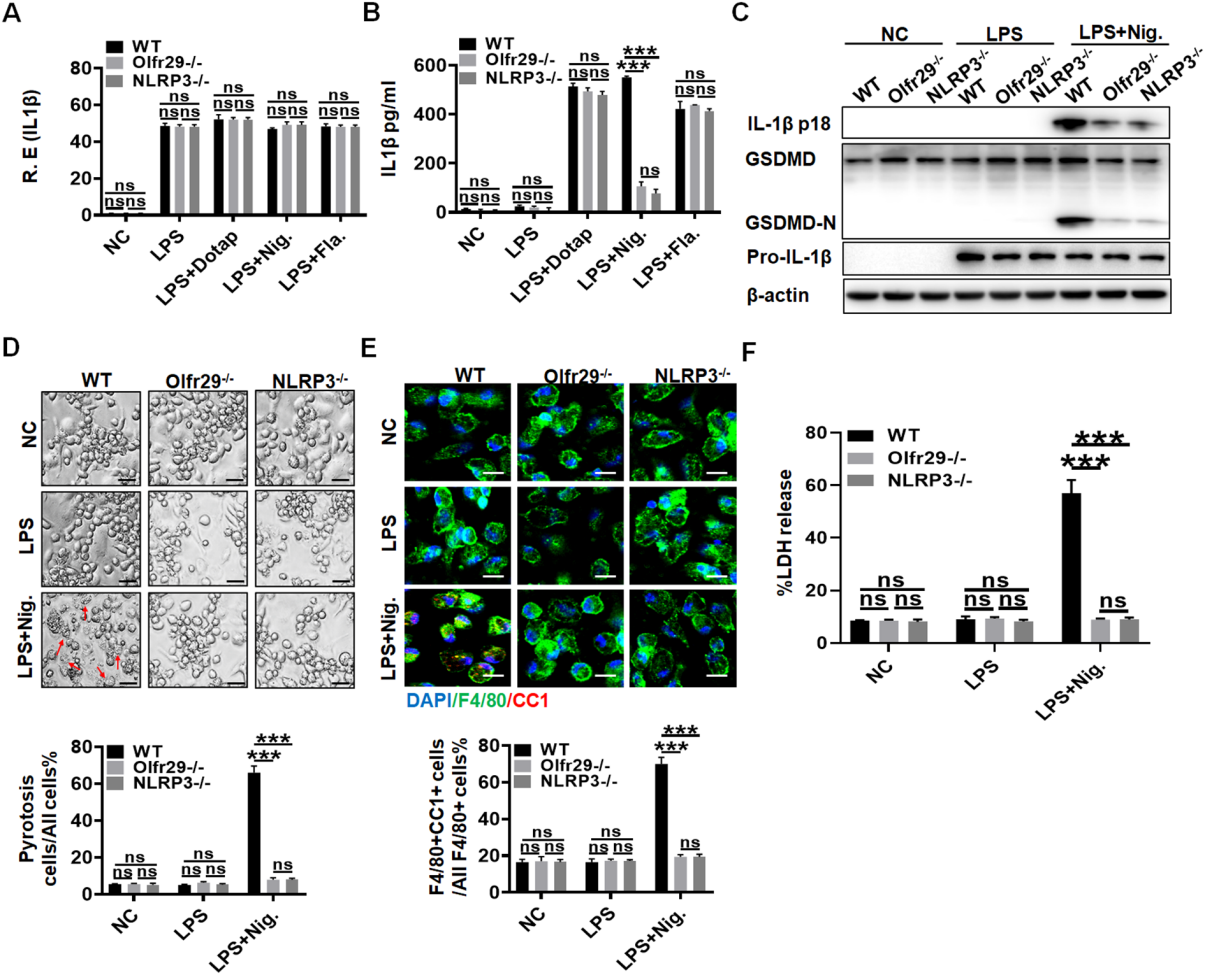
LncOlfr29 and NLRP3 KO mice have similar functions. **(A)** QRT-PCR of IL-1β in the macrophages of lncOlfr29 -/- (Olfr29 -/-), NLRP3 -/- and WT mice. **(B)** ELISA of IL-1β in the supernatants of the macrophages from lncOlfr29 -/- (Olfr29 -/-), NLRP3 -/- and WT mice. **(C)** Immunoblotting of mature IL-1β and GSDMD in the macrophages of lncOlfr29 -/- (Olfr29 -/-), NLRP3 -/- and WT mice. **(D)** Observation under the light microscope for the macrophages from lncOlfr29 -/- (Olfr29 -/-), NLRP3 -/- and WT mice. Scale bar, 10 μm. Arrows, typical pyroptosis cells. **(E)** Immuno-staining of cleaved caspase-1 (CC-1) in the macrophages of lncOlfr29 -/-(Olfr29 -/-), NLRP3 -/- and WT mice. Scale bar, 25 μm. **(F)** LDH in the supernatants of macrophages from lncOlfr29 -/- (Olfr29 -/-), NLRP3 -/- and WT mice. The macrophages were isolated from the peritoneal cavity of thioglycollate-treated mice, and stimulated by LPS, LPS + Dotap, LPS + nigericin (Nig.) or LPS + flagellin (Fla.). NC, medium only. Data were shown by mean ± SD. Student’s *t*-test. Ns, no significance; ***p<0.001.

To further determine the functions of lncOlfr29, we generated lncOlfr29 KO mice (lncOlfr29 -/-). Since lncOlfr29 transfected or silenced macrophages exhibited difference in mature IL-1β production and pyroptosis only after exposed to NLRP3 ligand LPS with nigericin, this suggests that lncOlfr29 mediated mature IL-1β and pyroptosis might be through NLRP3. Thus, we also observed whether there existed similar roles between lncOlfr29 and NLRP3. Interestingly, while lncOlfr29 KO and NLRP3 KO macrophages were exposed to LPS, LPS plus DOTAP, LPS plus nigericin and LPS plus flagellin, they exhibited similar results. Both lncOlfr29 -/- mice and NLRP3 -/- macrophages exhibited reduced mature IL-1β but not IL-1β mRNA upon exposure to NLRP3 ligands LPS plus nigericin as compared to the controls (**Figures 3A–C**). LncOlfr29 -/- and NLRP3 -/- macrophages also exhibited resistance to LPS plus nigericin mediated pyroptosis (**Figure 3D**). Consistent with LPS plus nigericin mediated pyroptosis, the number of CC1+ macrophages was significantly reduced in both lncOlfr29 -/- and NLRP3 -/- macrophages as compared to controls (**Figure 3E**). Whereas, there was no difference between lncOlfr29 -/- and NLRP3 -/- macrophages (**Figures 3A–E**). Finally, the release of lactate dehydrogenase (LDH) also decreased significantly in both lncOlfr29 -/- and NLRP3 -/- macrophages as compared to control macrophages (**Figure 3F**). Again, there are no difference between lncOlfr29 -/- and NLRP3 -/- macrophages (**Figure 3F**). Thus, similar to NLRP3, lncOlfr29 can promote pyroptosis of macrophages and production of mature IL-1β.

### 3.4 LncOlfr29 promotes resistance to Salmonella infection

**Figure 4:**
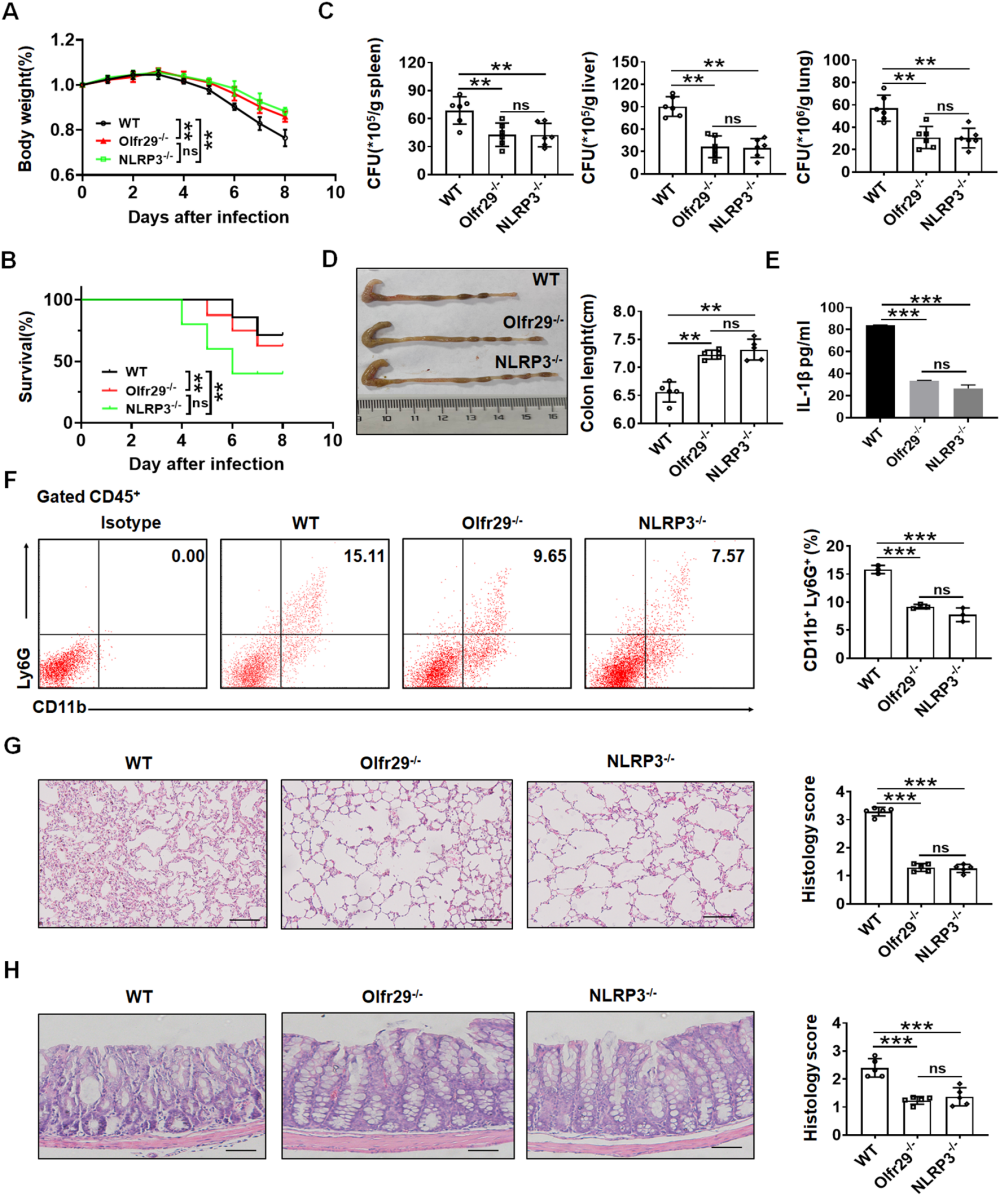
LncOlfr29 promotes resistance to Salmonella infection. **(A)** Body weight of lncOlfr29 -/- (Olfr29 -/-), NLRP3 -/- and WT mice after S. T infection. **(B)** Mortality rate of lncOlfr29 -/- (Olfr29 -/-), NLRP3-/- and WT mice after S. T infection. **(C)** The number of S.T clones in spleen, liver and lung tissues in lncOlfr29 -/- (Olfr29 -/-), NLRP3-/- and WT mice after S. T infection. **(D)** The colon length of mice in lncOlfr29 -/- (Olfr29 -/-), NLRP3-/- and WT mice after S. T infection. **(E)** ELISA of IL-1β in the serum of lncOlfr29 -/- (Olfr29 -/-), NLRP3-/- and WT mice after S. T infection. **(F)** Flow cytometry of CD11b (+) Ly6G (+) neutrophils in the lamina propria of the colon of lncOlfr29 -/- (Olfr29 -/-), NLRP3 -/- and WT mice after S. T infection. **(G)** H/E staining of the lung tissue in the lncOlfr29 -/- (Olfr29 -/-), NLRP3 -/- and WT mice after S. T infection. Scale bar, 45 μm. **(H)** H/E staining of the colon tissue in the lncOlfr29 -/- (Olfr29 -/-), NLRP3 -/- and WT mice after S. T infection. Scale bar, 45 μm. Data were shown by mean ± SEM; Analysis of variance test in **(A)**; Wilcoxon’s test in **(B)**; Two side Student’s *t*-test in **(C–H)**; Ns, no significance; **p<0.01;***p<0.001.

Previous studies showed that NLRP3 played an important role to Salmonella infection (33–35). Since lncOlfr29 -/- and NLRP3 -/- macrophages had similar functions, we next used a mouse model of S. T infection to test the effect of lncOlfr29. Similar to NLRP3 -/- mice, lncOlfr29 -/- mice had also reduced resistant to Salmonella infection. Both Salmonella-infected lncOlfr29 -/- and NLRP3 -/- mice had less weight loss and lower mortality rates as compared to wild type (WT) mice (**Figures 4A, B**). However, there were not different in body weight loss and mortality between lncOlfr29 -/- and NLRP3 -/- mice. Since Salmonella could invade organs other than the intestinal tract, such as the liver, spleen and lung tissues, we also detected the number of Salmonella in these organ tissues. The number of S. T bacteria in lncOlfr29 -/- and NLRP3 -/- mice was significantly lower than those in WT mice (**Figure 4C**). Again, S. T numbers were no differences in these organs between lncOlfr29-/- and NLRP3 -/- mice (**Figure 4C**). The colon lengths of lncOlfr29 -/- and NLRP3 -/- mice were also longer than those of WT mice (**Figure 4D**), indicating that lncOlfr29 and NLRP3 KO can alleviate intestinal inflammation. The inflammatory cytokine IL-1β in lncOlfr29 -/- was also significantly lower than that in WT mice (**Figure 4E**). We also analyzed the inflammatory cells in the lamina propria of the colon of mice. The results indicated that the proportion of neutrophils in lncOlfr29 -/- and NLRP3 -/- mice was less than that in WT mice (**Figure 4F**). H/E staining also showed that the inflammation in lung tissue was significantly reduced in lncOlfr29 -/- mice as compared to control, consistent with that in NLRP3 -/- mice (**Figure 4G**). The inflammation in the colon of lncOlfr29 -/- and NLRP3 -/- mice was also decreased as compared to WT mice (**Figure 4H**). Similar results were also found in S. T mediated acute infection model (**Supplementary Figure 3**). Therefore, similar to NLRP3, lncOlfr29 promotes resistance to Salmonella infection.

### 3.5 LncOlfr29 promotes sensitive to DSS-mediated colitis

**Figure 5:**
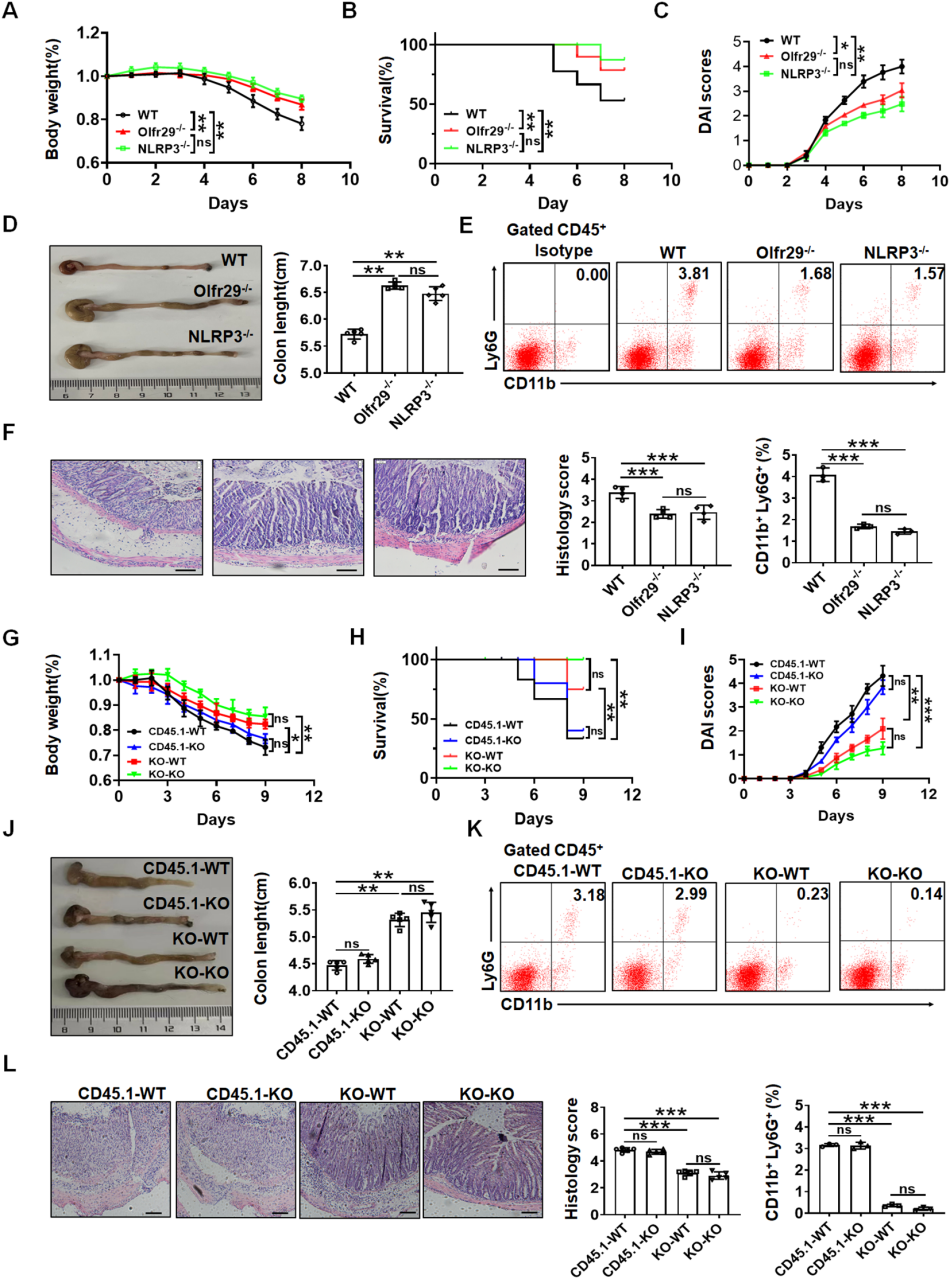
LncOlfr29 promotes sensitive to DSS-mediated colitis. **(A)** Body changes of lncOlfr29 -/- (Olfr29), NLRP3 -/- and WT mice after DSS (n=12). **(B)** Mortality rate of lncOlfr29 -/- (Olfr29 -/-), NLRP3 -/- and WT mice after DSS. **(C)** DAI of lncOlfr29 -/- (Olfr29 -/-), NLRP3 -/- and WT mice after DSS. **(D)** Colon length of lncOlfr29 -/- (Olfr29 -/-), NLRP3 -/- and WT mice after DSS. **(E)** Flow cytometry analysis of CD11b (+) Ly6G (+) in the lamina propria of the colon of lncOlfr29 -/- (Olfr29 -/-), NLRP3 -/- and WT mice after DSS. **(F)** H/E staining of lncOlfr29 -/- (Olfr29 -/-), NLRP3 -/- and WT mice after DSS. Scale bar, 45 μm. **(G)** Weight changes in macrophages transplanted mice after DSS. **(H)** Mortality rate in macrophages transplanted mice after DSS. **(I)** DAI in macrophages transplanted mice after DSS. **(J)** Colon length in macrophages transplanted mice after DSS. **(K)** Flow cytometry analysis of CD11b (+) Ly6G (+) in the lamina propria of the colon in macrophages transplanted mice after DSS. **(L)** H/E staining of colon tissues from macrophages transplanted mice after DSS. Scale bar, 45 μm. Data were shown by mean ± SEM. Macrophages transplanted mice were generated by transplanting macrophages from WT CD45.1 or lncOlfr29-/- mice into irradiated WT or lncOlfr29 -/- mice in G-L. CD45.1-WT, macrophages from CD45.1 mice were transplanted into WT mice; CD45.1-KO, macrophages from CD45.1 mice were transplanted into lncOlfr29 -/- mice; KO-WT, macrophages from lncOlfr29 -/- mice were transplanted into WT mice; KO-KO, macrophages from lncOlfr29 -/- mice were transplanted into lncOlfr29 -/- mice. Data were shown by mean ± SEM; Analysis of variance test in **(A, C, G, I)**; Wilcoxon’s test in **(B, H)**; Two side Student’s *t*-test in others. Ns, no significance; *p<0.05;**p<0.01;***p<0.001.

Macrophages are essential for the maintenance of intestinal homeostasis, and yet appear to be drivers of inflammation in the context of IBD (36, 37). Next, we further investigated the effects of lncOlfr29 on colitis using a mouse model of DSS-mediated colitis. DSS was administered for 7 days and normal water was given for 3 days to induce obvious symptoms of acute colitis in the mice. The results demonstrated that there had less weight loss and lower mortality rates in both lncOlfr29 -/- and NLRP3 -/- mice as compared to the WT mice (**Figures 5A, B**). Disease activated index (DAI) in lncOlfr29 -/- and NLRP3 -/- mice was also lower than WT mice (**Figure 5C**). The colon lengths of lncOlfr29 -/- and NLRP3 -/- mice were longer than those of WT mice, and the cecum was larger, indicating that the condition of intestinal inflammation has lessened (**Figure 5D**). The proportion of neutrophils in lncOlfr29 -/- and NLRP3 -/- mice also was lower than that those in WT mice (**Figure 5E**). The inflammatory courses of the colon in lncOlfr29 -/- and NLRP3 -/- mice were mush lighter than those in WT mice (**Figure 5F**). Notably, there were not different in DSS-mediated colitis between lncOlfr29 -/- and NLRP3 -/- mice. Thus, similar to NLRP3, lncOlfr29 promotes sensitivity to DSS-induced colitis.

LncOlfr29 is expressed not only in macrophages but also in other immune cells and intestinal epithelial cells. In order to eliminate the influence of lncOlfr29 in other immune cells and intestinal epithelial cells on colitis, the macrophage transplantation experiment was conducted. Bone marrow derived monocytes/macrophages were isolated from lncOlfr29 -/- and WT mice, and then injected via the tail vein into radiation-exposed mice. After receiving monocytes/macrophages from WT mice, there was no difference in DSS-induced colitis in lncOlfr29 -/- and WT mice (**Figures 5G–L**); There was also no difference in DSS-induced colitis in lncOlfr29 -/- and WT mice after receiving the macrophages from lncOlfr29 -/- mice (**Figures 5G–L**). Whereas there were significant differences in WT mice that received mononuclear/macrophages derived from WT or lncOlfr29 -/- mice, and lncOlfr29 -/- mice that received macrophages from WT or lncOlfr29 -/- mice (**Figures 5G–L**). Therefore, these results indicate that lncOlfr29 - mediated colitis is macrophage-dependent.

### 3.6 LncOlfr29 promotes NLRP3 expression by binding to ZFP36

**Figure 6:**
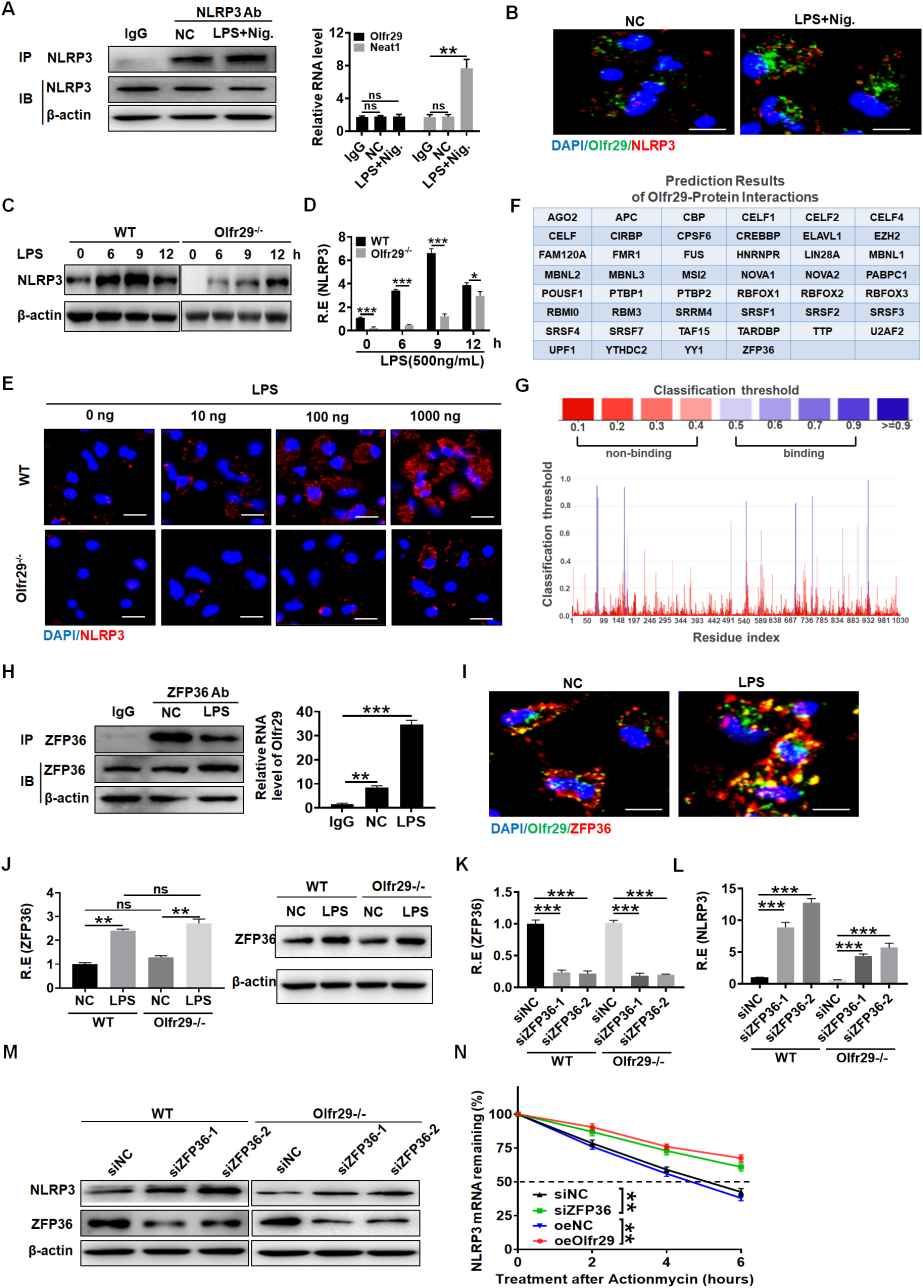
LncOlfr29 promotes NLRP3 expression by binding to ZFP36. **(A)** RIP in the macrophages after exposed to LPS + nigericin (Nig.). Cell lysates were incubated with NLRP3 antibody (NLRP3 Ab) or control rabbit IgG. The immunoprecipitates were analyzed by qRT-PCR to exam enrichment efficiency of LncOlfr29. Lnc-Neat1 (Neat1) was used as a positive control for binding to NLRP3. **(B)** RNA-FISH of NLRP3 and lncOlfr29 in the macrophages upon exposure to LPS + nigericin (Nig.). Red, NLRP3; Green, lncOlfr29 (Olfr29); Blue, nuclei. Scale bar, 2.5 μM. **(C)** Immunoblotting of NLRP3 in the macrophages of lncOlfr29 -/- (Olfr29 -/-) and WT mice with or without LPS stimulation. **(D)** QRT-PCR of NLRP3 in the macrophages of lncOlfr29 -/- (Olfr29 -/-) and WT mice with or without LPS stimulation. **(E)** Immunostaining of NLRP3 in the macrophages of lncOlfr29 -/- (Olfr29 -/-) and WT mice with or without LPS stimulation. Scale bar, 2.5 μm. **(F, G)** Prediction of the binding of lncOlfr29 (Olfr29) to ZFP36 **(F)** and the potential binding sites **(G, H)** RIP in the macrophages after exposed to LPS. Cell lysates were incubated with ZFP36 antibody (ZFP36 Ab) or control rabbit IgG. The immunoprecipitates were analyzed by qRT-PCR to exam enrichment efficiency of LncOlfr29 (Olfr29). **(I)** RNA-FISH of ZFP36 and lncOlfr29 (Olfr29) in macrophages. Red, ZFP36; Green, LncOlfr29; Blue, nuclei. Scale bar, 2.5 μM. **(J)** QRT-PCR and immunoblotting of ZFP36 in the WT and lncOlfr29 KO macrophages upon exposure to LPS. **(K–M)** QRT-PCR **(K, L)** and immunobotting **(M)** of ZFP36 **(K)** and NLRP3 **(L)** in WT and lncOlfr29 KO macrophages after silencing ZFP36 (siZFP36–1 or siZFP36-2). Data were shown by mean ± SD. **(N)** Actinomycin D chase assays of NLRP3 mRNA by time-course qPCR under conditions of ZFP36 depletion (siZFP36) or lncOlfr29 overexpression (oeOlfr29). Analysis of variance test in **(N)** ShNC, shRNA control; OeNC, oeOlfr29 control. R.E, relative expression. Macrophages transfected with ZFP36 siRNA (siZFP36) and exogenous lncOlfr29 (oeOlfr29) were treated with actinomycin D (0.5 mg/mL) for indicated time periods to initiate the time-course experiment. NLRP3 mRNA levels were detected by qRT-PCR. Data are presented as mean ± SD from three independent experiments. Two side student’s *t*-test. Ns, no significance; *p<0.05;**p<0.01;***p<0.001.

Since the function of lncOlfr29 was similar to that of NLRP3, we believed that the function of lncOlfr29 was achieved through NLRP3. We next looked for the mechanism(s) by which lncOlfr29 could promote activation of NLRP3. LncRNA can directly bind to proteins (13). Thus, we first analyzed the potential binding proteins of lncOlfr29. However, data did not show the binding of lncOlfr29 to NLRP3 (**Figure 6F**). Indeed, while the positive control lncRNA Neat1 (38) could bind to NLRP3, RIP did not show binding of NLRP3 with lncOlfr29 (**Figure 6A**). RNA immunofluorescence *in situ* hybridization (FISH) also did not exhibit the binding of lncOlfr29 with NLRP3 (**Figure 6B**). All of these suggest that lncOlfr29 has no direct binding to NLRP3. LncOlfr29 can also encodes polypeptides that have an impact on the cellular function. So we also analyzed whether the lncOlfr29 could encode polypeptide(s). The prediction results showed that lncOlfr29 did not encode any polypeptides (**Supplementary Figure 4**).

Next, we studied the effect of lncOlfr29 on the expression of NLRP3. Interestingly, the expression of NLRP3 in lncOlfr29 KO macrophages was lower than that in WT macrophages regardless of whether they were exposed to LPS (**Figure 6C**). QPCR and immunofluorescence assays also showed a reduction of NLRP3 in lncOlfr29 KO macrophages compared to WT macrophages (**Figures 6D, E**). The expression of NLRP3 can be regulated by various factors such as ZFP36, which can bind to the 3 ‘-UTR of mRNA to degrade NLRP3 mRNA (16, 39–41). Thus, we investigated whether lncOlfr29 could bind to ZFP36 to eliminate the degradation of NLRP3 mRNA by ZFP36. To do this, we again analyzed the potential binding protein of lncOlfr29. The prediction results show that there were multiple binding sites between lncOlfr29 and ZFP36 (**Figures 6F, G**). RIP experiment also showed a binding between lncOlfr29 and ZFP36 (**Figure 6H**). RNA-FISH analysis confirmed that lncOlfr29 could bind to ZFP36 (**Figure 6I**). These results suggest that lncOlfr29 can bind to ZFP36 and remove the degradation of NLRP3mRNA 3’UTR by ZFP36. Indeed, silencing ZFP36 not only upregulated protein but also mRNA levels of NLRP3 (**Figures 6J–M**). Critically, actinomycin D chase assays exhibited increased NLRP3 mRNA via time-course qPCR under conditions of ZFP36 silencin or lncOlfr29 overexpression (**Figure 6N**). Taken together, lncOlfr29 can combine with ZFP36 to promote NLRP3 expression.

### 3.7 Function of human lncOlfr29 is similar to that of mice

Finally, we investigated whether the biological functions of human lncOlfr29 (hulncOlfr29) in macrophages were similar to those in mice.

**Figure 7:**
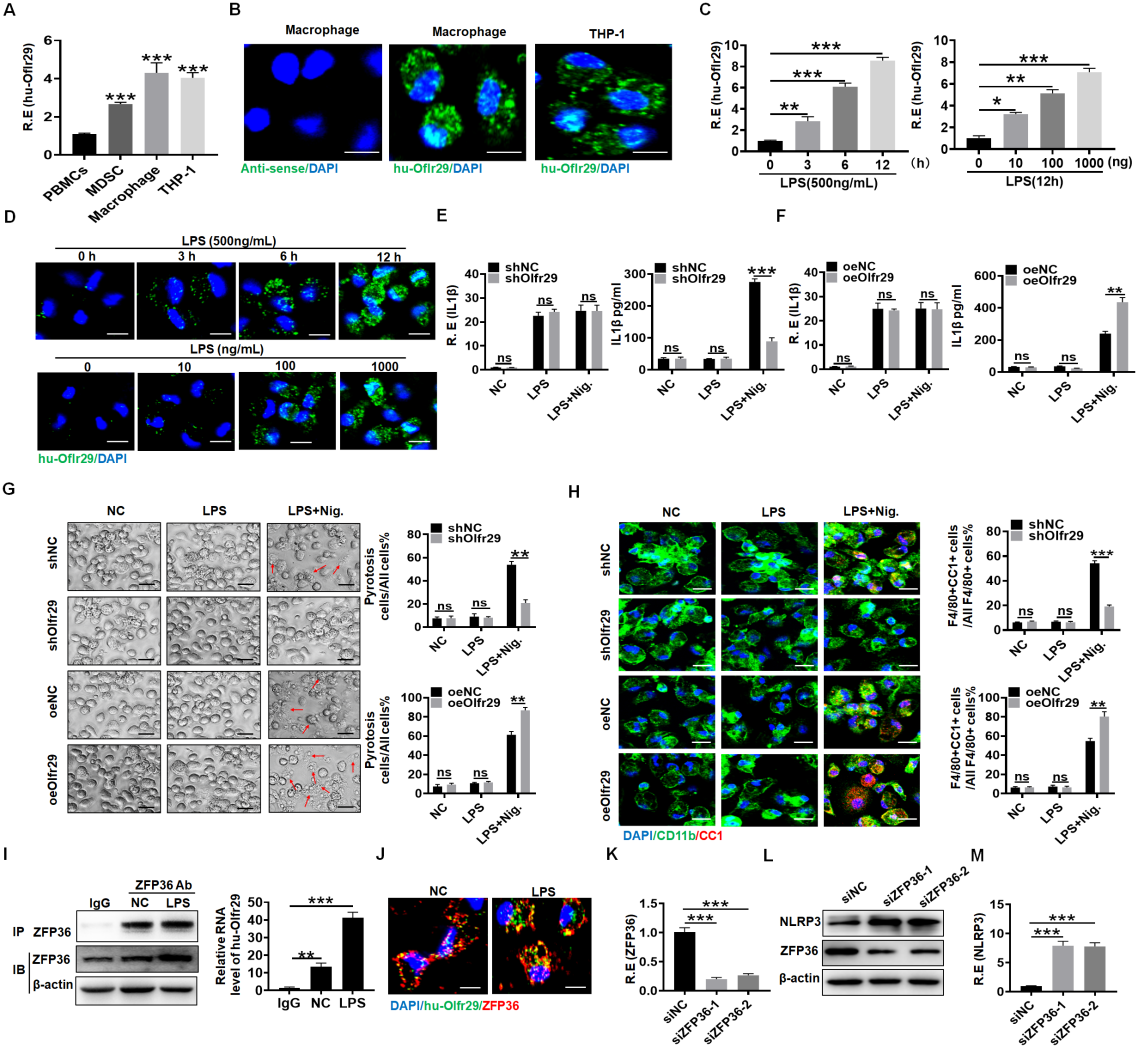
Function of human lncOlfr29 is similar to that of mice. **(A, B)** QRT-PCR and RNA-FISH of human lncOlfr29 (huOlfr29) in peripheral blood monocytes (PBMCs), MDSCs, macrophages and human macrophage cell line THP-1. Scale bar, 2.5 μM. **(C)** QPCR of huOlfr29 in the human macrophages upon exposure to LPS at different times and concentrations. **(D)** RNA-FISH of huOlfr29 in the human macrophages upon exposure to LPS at different times and concentrations. Scale bar, 2.5 μM. **(E)** QRT-PCR and ELISA of IL-1β in the human lncOlfr29 shRNA (shOlfr29) transfected macrophages. **(F)** QRT-PCR and ELISA of IL-1β in the human exogenous lncOlfr29 (oeOlfr29) transfected macrophages. **(G)** Observation under light microscope for the human shOlfr29 and oeOlfr29 transfected macrophages after 24 hrs. Scale bar, 45 μM. Arrows, typical pyroptosis cells. **(H)** Immunostaining of cleaved caspase-1 (CC-1) in the human shOlfr29 and oeOlfr29 transfected macrophages after 24 hrs. Scale bar, 2.5 μM. **(I)** RIP in macrophages after exposed to LPS. Cell lysates were incubated with ZFP36 antibody (ZFP36) or control rabbit IgG. The immunoprecipitates were analyzed by qRT-PCR to exam enrichment efficiency of lncOlfr29. **(J)** RNA-FISH of ZFP36 and huOlfr29 in macrophages. Red, ZFP36; Green, LncOlfr29; Blue, nuclei. Scale bar, 2.5 μM. **(K)** QRT-PCR of ZFP36 in the macrophages after silencing ZFP36. **(L, M)** Immunobotting and qRT-PCR of NLRP3 in the macrophages after silencing ZFP36 (siZFP36–1 or siZFP36-2). Data were shown by mean ± SD. Two side Student’s *t*-test. Ns, no significance; *p<0.05;**p<0.01;***p<0.001.

The expression of human lncOlfr29 in macrophages and the macrophage cell line THP1 could also be detected (**Figures 7A, B**). Furthermore, LPS also promoted expression of human lncOlfr29 in macrophages (**Figure 7C**). RNA-FISH also showed increased expression of human lncOlfr29 in human macrophages after exposure to LPS (**Figure 7D**). Importantly, silencing human lncOlfr29 could not only reduce production of mIL-1β but also pyroptosis of human macrophages upon exposure to NLRP3 ligand LPS plus nigericin; whereas overexpressed hulncOlfr29 increased the production of mIL-1β and pyroptosis of macrophages (**Figures 7E–H**). In terms of mechanism, ZFP36 also combined with hulncOlfr29 (**Figures 7I, J**). Silencing ZFP36 also promoted the expression of NLRP3 in human macrophages (**Figures 7K–M**). Therefore, human lncOlfr29 has similar functions to mouse lncOlfr29 in macrophages.

## 4 Discussion

**Figure 8:**
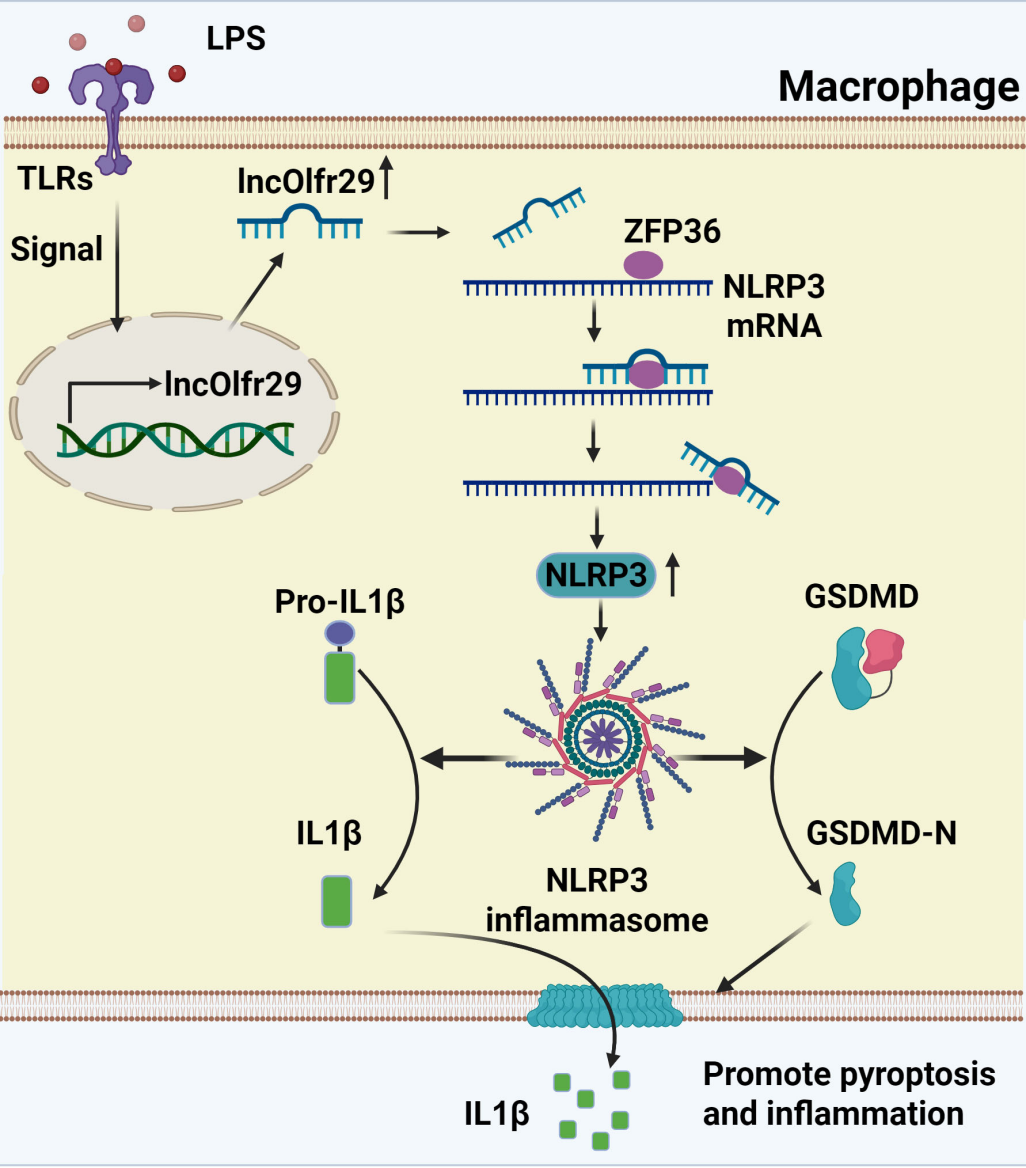
A mechanistic schematic explicitly. LncRNA lncOlfr29 can control the inflammatory response of macrophages through the Olfr29/ZFP36/NLRP3 axis. LncOlfr29 promotes the expression of NLRP3 by binding to ZFP36, eliminating the degradation of NLRP3 mRNA by ZFP36.

We here found that lncOlfr26 can control the inflammatory response of macrophages. LncOlfr29 promotes the expression of NLRP3 by binding to ZFP36, eliminating the degradation of NLRP3 mRNA by ZFP36 (**Figure 8**). Indeed, knockout of lncOlfr29 leads to decreased expression of NLRP3, reduced pyroptosis of macrophages and decreased maturation of IL-1β, ultimately inhibiting the development of inflammation. Importantly, lncOlfr29 exhibits similar biological functions in human and mouse macrophages. These results will provide new insights into the treatment of inflammatory diseases.

LncOlfr29 is a potential target for the immunotherapy of inflammation associated diseases. Macrophages are almost universally present in various tissues in different environments (42). These macrophages are crucial cells in the innate immune system, contributing to maintenance of tissue development and homeostasis, clearance host defense during pathogen infection, and promotion of tissue repair in response to tissue injury (43, 44). They are closely associated with the development of various diseases such as inflammation associated diseases (1–3). Our results demonstrate that lncOlfr29 can promote inflammatory responses of macrophages, suggesting that lncOlfr29 can be a potential target for these diseases. Targeting lncRNAs have become an attractive approach for treating various diseases (45).

We demonstrate that lncOlfr29 can promote inflammation through binding with ZFP36. The mRNA-destabilizing ZFP36 is a major driver of the inflammatory gene expression control. It is RNA-binding proteins involved in mRNA metabolism pathways (39). ZFP36 which contains two tandemly repeated CCCH-type zinc-finger motifs, can bind to adenine uridine-rich elements in the 3’-untranslated regions (3’ UTR) of specific mRNA, and lead to target mRNA decay (39, 41). Previous study showed that ZFP36 is a negative regulatory factor of NLRP3 in macrophages through acts on the 3’-UTR region of NLRP3 and ultimately inhibits the expression of NLRP3. Knockout of ZFP36 in macrophages also leads to an increase of NLRP3 (16, 46, 47). However, it remains unclear how to control the influence of ZFP36 on the expression of NLRP3. This study indicates that lncOlfr29 can bind to the ZFP36 protein and reduce degradation of NLRP3 mRNA in macrophages, suggesting a novel post-transcriptional mechanism regulating NLRP3 expression. The binding of lncOlfr29 to ZFP36 can reduce the degradation of NLRP3mRNA by excluding the binding of ZFP36 to the promoter region of NLRP3mRNA, thereby promoting an increase in NLRP3 expression. Notably, several studies have investigated the regulatory role of lncRNAs in modulating NLRP3 expression and function (38, 48, 49). For instance, a recent study found that LncRNA LINC00969 promotes acquired gefitinib resistance in lung cancer by epigenetically suppressing NLRP3, thereby inhibiting pyroptosis (50).

We also demonstrate that lncOlfr29 expression can be rapidly induced by LPS. Notably, lncOlfr29 expression is also potentially affected by other factors such as pathogen exposure and microbiota composition. Since NLRP3 activation generally needs a longer time (51–53), the rapid expression of LPS-mediated lncOlfr29 may have an important significance in switching or promoting the activation of NLRP3 by infection to induce inflammation and eliminate bacteria.

## Data Availability

The original contributions presented in the study are included in the article/**Supplementary Material**. Further inquiries can be directed to the corresponding authors.
