# Supplementary material for: Regulation of ZFP36 by lncOlfr29 promotes inflammation through NLRP3
Source: Front Immunol. 2025 Aug 26;16:1642783. doi: 10.3389/fimmu.2025.1642783 (PMC12417172; doi:10.3389/fimmu.2025.1642783)
Supplement: Supplementary file 2 [file DataSheet2.docx]

**Supplementary information:**

**Regulation of ZFP36 by lncOlfr29 promotes inflammation through NLRP3**

Wenyue Cheng^1^, Fan Li^1^, Yuan Zhang^1^_,_ Yunhuan Gao^1^, and Rongcun Yang^1,2,3^

**
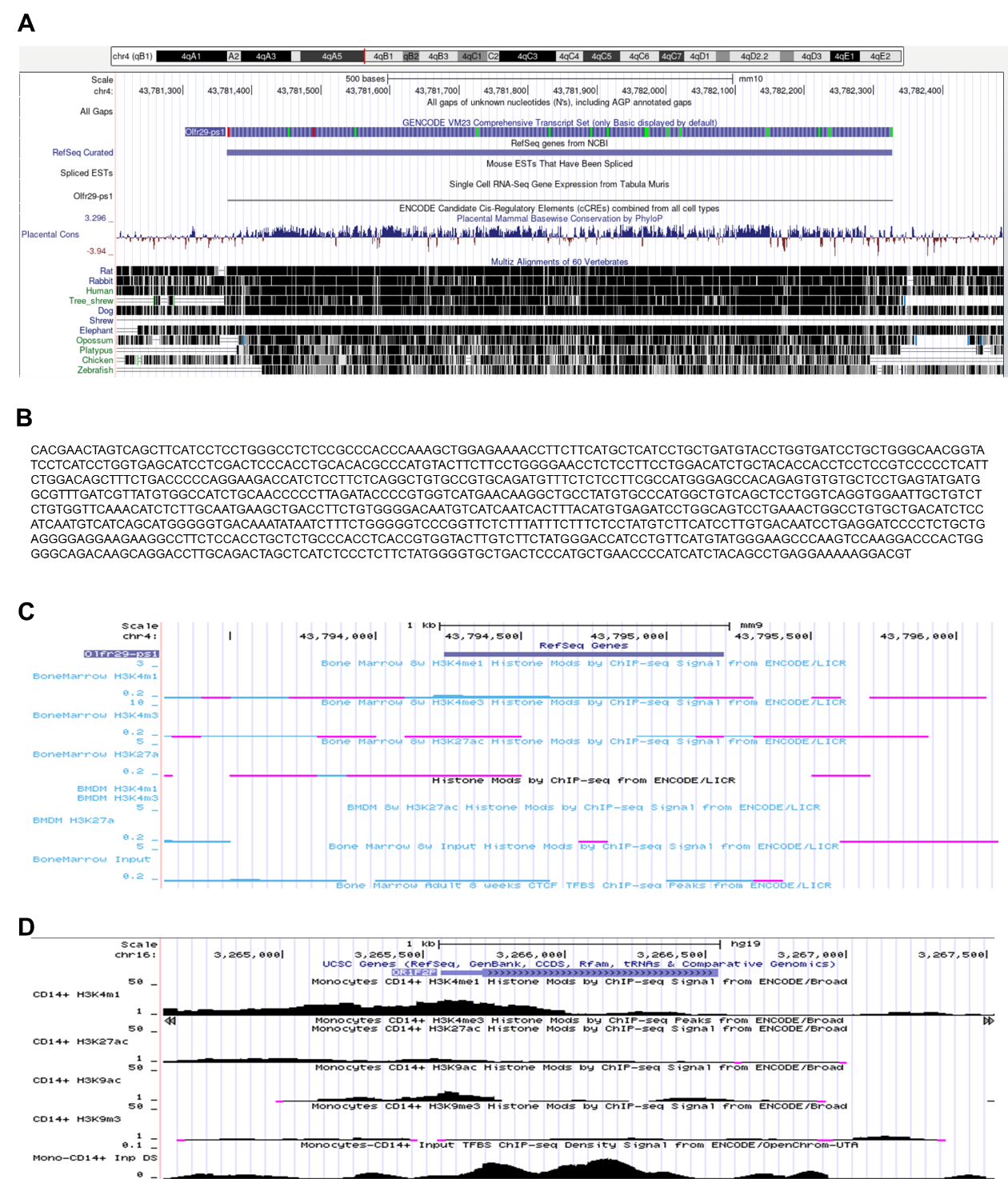
**

**Supplementary Figure 1.** Characteristics of lncOlfr29. **A.** Genome Browser image of Olfr29-ps1 location in mice chromosome (mm10). **B.** Sequences of mouse lncRNA Olfr29-ps1. **C.** Genome Browser image showing the distribution of RNA reads and the distribution of H3K4me1, H3K4me3, H3K27ac in mouse myeloid derived cells. **D.** Genome Browser image showing the distribution of RNA reads and the distribution of H3K4me1, H3K9ac, H3K9me3 and H3K27ac in human CD14^+^ myeloid-derived cells.

**
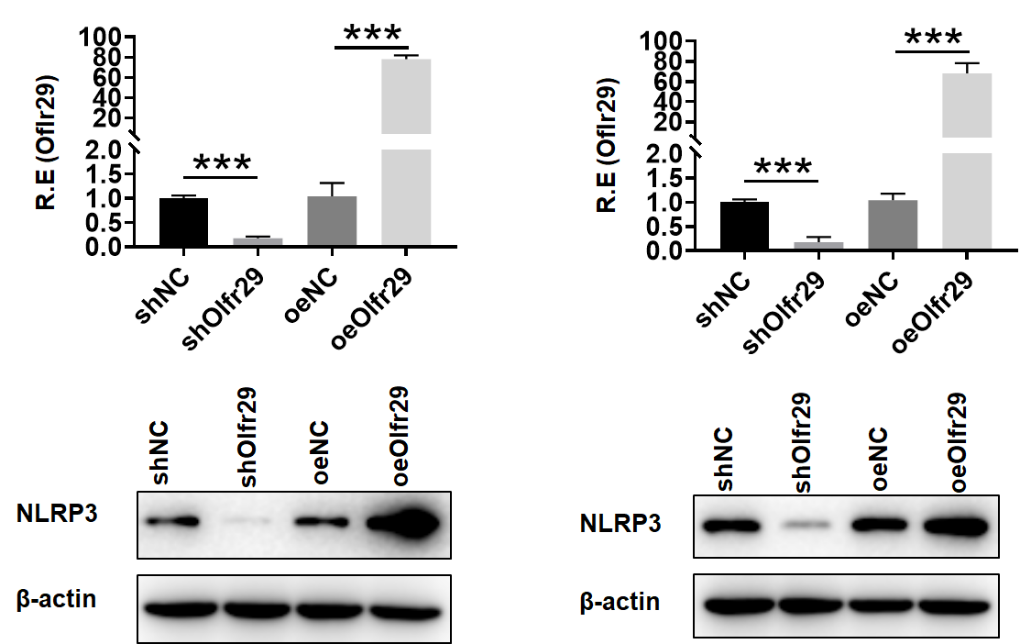
**

**Supplementary Figure 2.** The levels of lncOlfr29 and NLRP3 protein in lncOlfr29 silencing (shOlfr29) and overexpressing (oeOlfr29) macrophages. ShNC, shRNA control; OeNC, oeOlfr29 control. R.E, relative expression.

Data were shown by mean ± SEM;

Two side Student’s t-test; ***p<0.001.


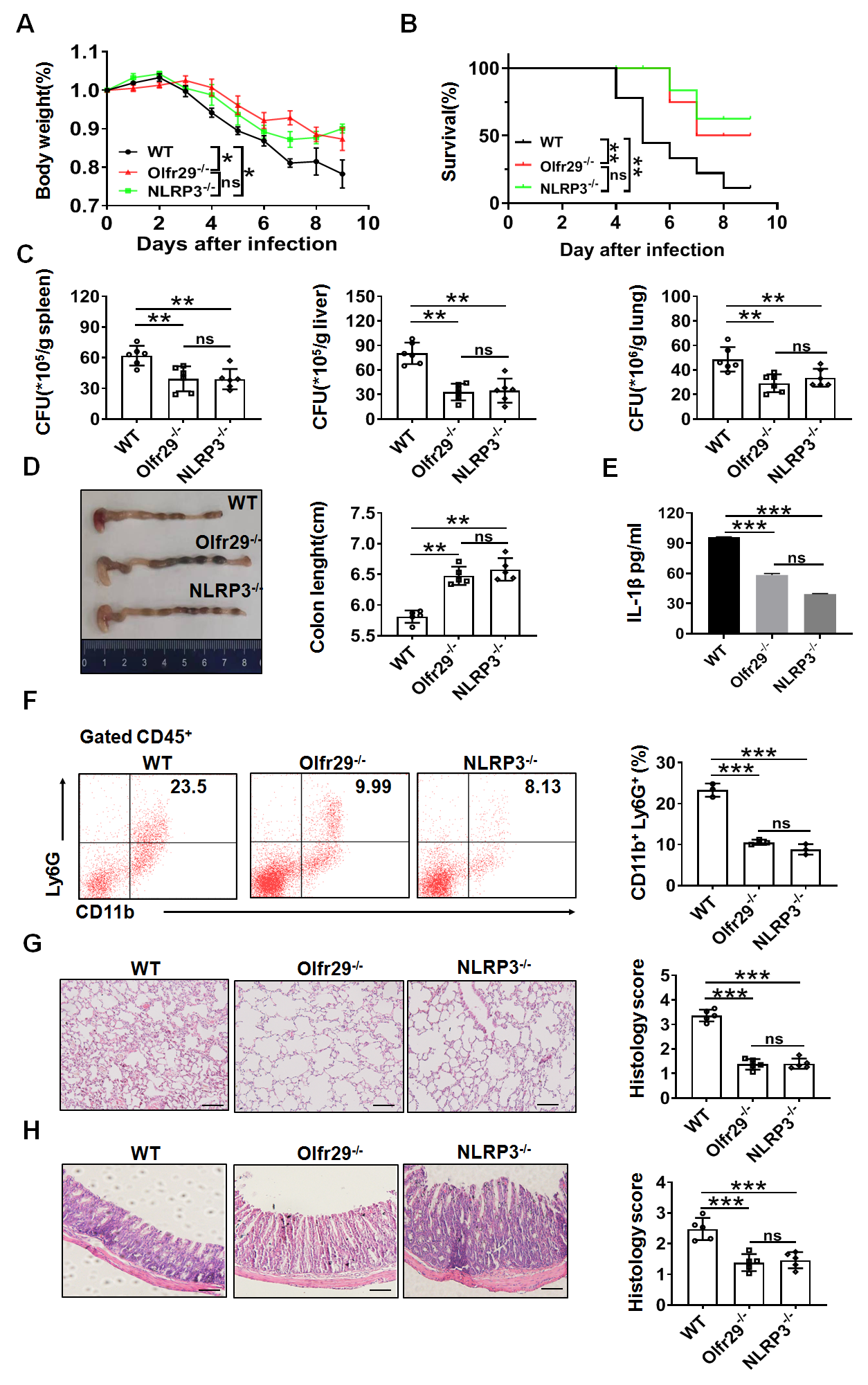


**Supplementary Figure 3.** LncOlfr29 promotes resistance to acute S. T infection. **A.** Body weight of LncOlfr29 -/- (Olfr29 -/-), NLRP3-/- and WT mice after S. T infection. **B.** Mortality rate of lncOlfr29-/-(Olfr29 -/-), NLRP3-/- and WT mice after S. T infection. **C.** The number of ST clones in spleen, liver and lung tissues in lncOlfr29-/-(Olfr29 -/-), NLRP3-/- and WT mice after S. T infection. **D.** The colon length in lncOlfr29-/- (Olfr29 -/-), NLRP3 -/- and WT mice after S. T infection. **E.** ELISA of IL-1β in the sera of mice in (A). **F.** Flow cytometry of CD11b(+)Ly6G (+) neutrophils in the lamina propria of the colon of lncOlfr29-/-(Olfr29 -/-), NLRP3-/- and WT mice after S. T infection. **G.** H/E staining of mouse lung tissue in the lncOlfr29 -/- (Olfr29 -/-), NLRP3-/- and WT mice after S. T infection. Scale bar, 45μM. **H.** H/E staining of colon tissue in the lncOlfr29-/-(Olfr29 -/-), NLRP3-/- and WT mice after S. T infection. Scale bar, 45μM.

Data were shown by mean ± SEM;

Analysis of variance test in A; Wilcoxon’s test in B; Two side Student’s t-test in C-H

Ns, no significance; *p<0.05；**p<0.01；***p<0.001.


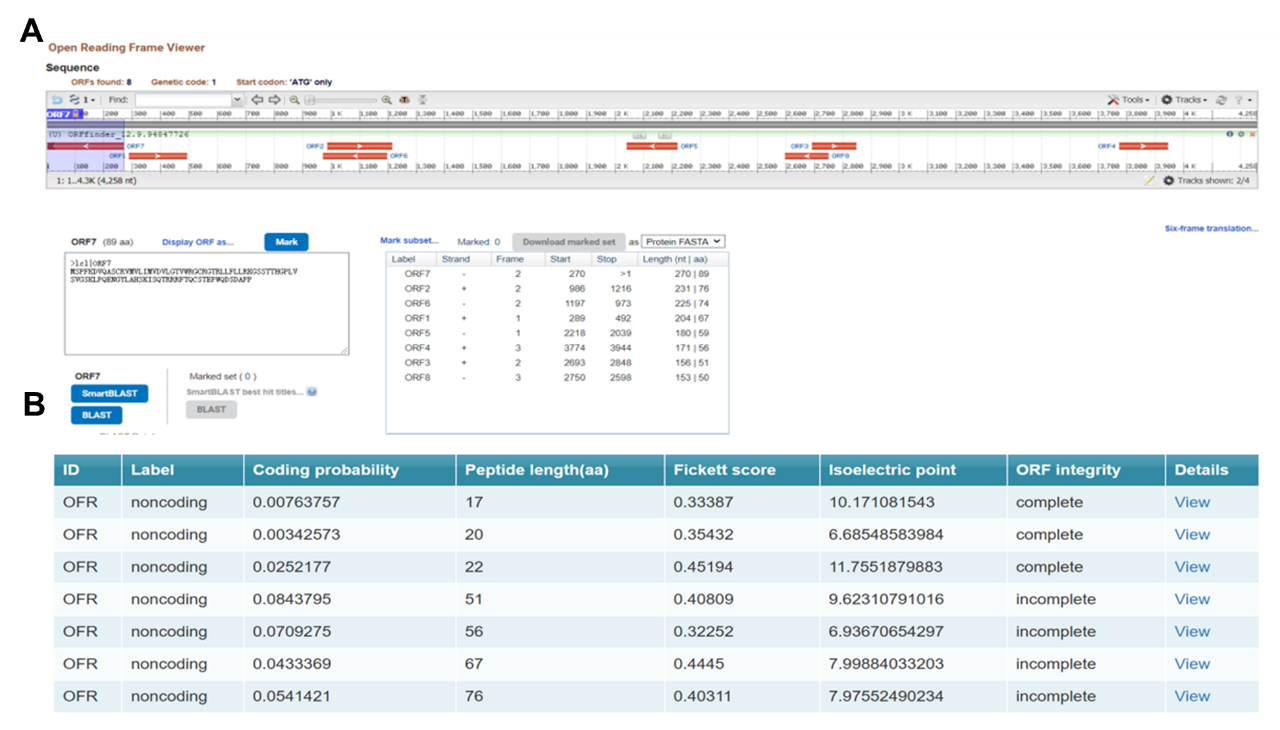


**Supplementary Figure 4.** LncOlfr29 does not encode the peptides.

1. Open reading frames on lncOLFR29 (https://www.ncbi.nlm.nih.gov/orffinder/). **B.** CPC2 analyses of the coding potential of ORFs in the lncOLFR29 (CPC2, http:/ /cpc2.gao-lab.org/).

**Supplementary Table 1. Reagents and oligoes used in this study.**

| REAGENT or RESOURCE | SOURCE | IDENTIFIER |
| --- | --- | --- |
| Antibodies | | |
| β-Actin Antibody | Santa Cruz | Cat:sc-47778 RRID: AB_626632 |
| ZFP36 (TTP) Polyclonal antibody | Proteintech | Cat: 12737-1-AP, RRID: AB_10598485 |
| Anti-NLRP3 antibody | Abcam | Cat: ab263899 RRID: AB_2889890 |
| Anti-GSDMD antibody | Abcam | Cat: ab209845 RRID: AB_2721254 |
| Anti -IL1β antibody | Proteintech | Cat: 16806-1-AP RRID: AB_10646432 |
| Anti- Cleaved Caspase-1 antibody | Cell Signaling Technology | Cat：67314 RRID: AB_2714037 |
| F4/80 (6A545) antibody | Santa Cruz | Cat:sc-71085 RRID: AB_1122717 |
| CD11B Polyclonal antibody | Proteintech | Cat: 21851-1-AP RRID: AB_2878927 |
| APC-CD45 30-F11 | Biolegend | Cat:103112 RRID: AB_312977 |
| FITC-CD11b M1/70 | eBioscience | Cat:11-0112-86 RRID: AB_464937 |
| PE-Ly6G 1A8 | Biolegend | Cat: 127607 RRID: AB_1186104 |
| Bacterial and Virus Strains | | |
| S. Typhimurium | ATCC | Cat: 14028 |
| Chemicals, Peptides, and Recombinant Proteins | | |
| Recombinant Murine GM-CSF | PeproTech | Cat:315-03 |
| Recombinant Murine M-CSF | PeproTech | Cat:315-02 |
| Recombinant Human M-CSF | PeproTech | Cat: 300-25 |
| Recombinant Human IFN-γ | PeproTech | Cat: 300-02 |
| Recombinant Murine IFN-γ | PeproTech | Cat: 315-05 |
| HiPerFect Transfection Reagent | QIAGEN | Cat:301705 |
| DOTAP chloride | Selleck | Cat: S6908 |
| LPS (0111:B4) | Sigma | Cat: L2630 |
| Nigericin | MedChemExpress | Cat: 28380-24-7 |
| Flagellin | AdipoGen Life Sciences | Cat: AG-40B-0095 |
| Deoxycholic acid (DCA) | Selleck | Cat: S4689 |
| Indole-3-acetic acid (IAA) | Selleck | Cat: S4799 |
| Phorbol 12-myristate 13-acetate (PMA) | selleck | Cat: S7791 |
| Actinomycin D | MedChemExpress | Cat: HY-17559 |
| Lipofectamine™ 3000 Transfection Reagent | Thermo Fisher Scientific | Cat:11668027 |
| Trizol | Life technologies | Cat:15596018 |
| Experimental Models: Cell Lines | | |
| HEK 293T | ATCC | N/A |
| THP-1 | ATCC | N/A |
| Oligonucleotides for qRT-PCR | | |
| Murine GAPDH FW | BGI | 5’-TCAACGGCACAGTCAAGG-3’ |
| Murine GAPDH REV | BGI | 5’-TACTCAGCACCGGCCTCA-3’ |
| Murine Olfr29-ps1 FW | BGI | 5’-TAACAACCAGACCTCGTTCCC-3’ |
| Murine Olfr29-ps1 REV | BGI | 5’-ATGAGGATACCGTTGCCCAG-3’ |
| Murine IL1β FW | BGI | 5’-TCGCAGCAGCACATCAACAAG-3’ |
| Murine IL1β REV | BGI | 5’-GAAGGTCCACGGGAAAGACAC-3’ |
| Murine Neat1 FW | BGI | 5’-GGCACAAGTTTCACAGGCCTAC-3’ |
| Murine Neat1 REV | BGI | 5’-GCCAGAGCTGTCCGCCCAGCGA-3’ |
| Murine NLRP3 FW | BGI | 5’-GAGTTCTTCGCTGCTATGTA-3’ |
| Murine NLRP3 REV | BGI | 5’-AGAGGTTCTCTCCTGGTTTA-3’ |
| Murine ZFP36 FW | BGI | 5’-TGTCCTCTTGTTCCTTTTCG-3’ |
| Murine ZFP36 REV | BGI | 5’-CCTGGTTAGGGTCTCTTCGA-3’ |
| Human GAPDH FW | BGI | 5’- TCAAGAAGGTGGTGAAGCAGG-3’ |
| Human GAPDH REV | BGI | 5’- AGCGTCAAAGGTGGAGGAGTG-3’ |
| Human OR1F2P FW | BGI | 5’-TGTGAGGTTCACAGTGGTGAC-3’ |
| Human OR1F2P REV | BGI | 5’-ACCACTTTTACAAGAGCCCCT-3’ |
| Human IL1β FW | BGI | 5’- GGCAATGAGGATGACTTGTTC-3’ |
| Human IL1β REV | BGI | 5’- TGCTGTAGTGGTGGTCGGAGA-3’ |
| Human NLRP3 FW | BGI | 5’- AGCCTCAACAAACGCTACAC -3’ |
| Human NLRP3 REV | BGI | 5’- ATCTTAATGGGACTCACGGG -3’ |
| Human ZFP36 FW | BGI | 5’- ACGGGGCCAAGTGCCAGTTT -3’ |
| Human ZFP36 REV | BGI | 5’- GCCAGGTCTTCGCTAGGGTT -3’ |
| Probes used in the RNA-FISH | | |
| Olfr29-5’FAM | BGI | 5’-TTCGTCACAGCAGCCCTCAC -3’ |
| OR1F2P-5’FAM | BGI | 5’-GACAGTGAATTCAGAAACATAC-3’ |
| Murine NLRP3-5’FAM | BGI | 5’-GCATTGCTTCGTAGATAGAGGT-3’ |
| Human NLRP3-5’FAM | BGI | 5’-CACTTCGGCTCATCTCTTTTTGC-3’ |
| NC-5’FAM | BGI | 5’CGGGAGCCTAGGAAGTGCATCT-3’ |
| siRNAs used in this study | | |
| Murine lncRNA Olfr29 | Ribobio | 5’-CCGUGGUACUUGUCUUCUAUG-3’ |
| Human lncRNA OR1F2P | Ribobio | 5’-CACUCAGCUCAGAGAGAUAUA-3’ |
| Murine ZFP36-1 | Ribobio | 5’-GGAGGACUUUGGAACAUAAAC-3’ |
| Murine ZFP36-2 | Ribobio | 5’-GAGCUGUCACCCUCACCUACU-3’ |
| Human ZFP36-1 | Ribobio | 5’-GCGCUACAAGACUGAGCUAUG-3’ |
| Human ZFP36-2 | Ribobio | 5’-AGACGGAACUCUGUCACAAGU-3’ |
| Other | Ribobio |  |
| Ampicillin | Sigma | Cat: BP021 |
| Vancomycine | Sigma | Cat: V2002 |
| DMEM | Gibco | Cat:11965118 |
| FBS | Gibco | Cat:10099141 |
| HBSS | Gibco | Cat:14170161 |
| pcDNA™3.1/V5-His TOPO® TA  Expression Kit | Invitrogen | Cat: K4800-40 |
| Pierce™ Protein G Agarose | Thermo Fisher Scientific | Cat: 20397 |
| LDH Assay Kit | Abcam | Cat: ab102526 |
| Mouse IL-1β ELISA Kit | ABclonal | Cat: RK04878 |
| Human IL-1 β ELISA Kit | ABclonal | Cat: RK00001 |
